# Expression and copy number analysis of TRPS1, EIF3S3 and MYC genes in breast and prostate cancer

**DOI:** 10.1038/sj.bjc.6601648

**Published:** 2004-03-02

**Authors:** K J Savinainen, M J Linja, O R Saramäki, T L J Tammela, G T G Chang, A O Brinkmann, T Visakorpi

**Affiliations:** 1 Cancer Genetics, Institute of Medical Technology, University of Tampere and Tampere University Hospital, FIN-33014 Tampere, Finland; 2 Department of Urology, University of Tampere and Tampere University Hospital, FIN-33014 Tampere, Finland; 3 Department of Reproduction and Development, Erasmus MC, 3000 DR Rotterdam, The Netherlands

**Keywords:** prostate cancer, breast cancer, TRPS1, EIF3S3, MYC, overexpression

## Abstract

The long arm of chromosome 8 is one of the most common regions of amplification in cancers of several organs, especially carcinomas of the breast and prostate. TRPS1, MYC and EIF3S3 genes are located in one of the minimal regions of amplification, 8q23–q24, and have been suggested to be the target genes of the amplification. Here, our goal was to study copy number and expression of the three genes in order to investigate the significance of the genes in breast and prostate cancer. By using fluorescence *in situ* hybridisation (FISH), we first found that TRPS1 and EIF3S3 were amplified together in about one-third of hormone-refractory prostate carcinomas. Next, we analysed the mRNA expression of the three genes by real-time quantitative RT–PCR and the gene copy number by FISH in six breast and five prostate cancer cell lines. Breast cancer cell line, SK-Br-3, which contained the highest copy number of all three genes, showed overexpression of only EIF3S3. Finally, the expression levels of TRPS1, EIF3S3 and MYC were measured in freshly frozen clinical samples of benign prostate hyperplasia (BPH), as well as untreated and hormone-refractory prostate carcinoma. The TRPS1 and MYC expression levels were similar in all prostate tumour groups, whereas EIF3S3 expression was higher (*P*=0.029) in prostate carcinomas compared to BPH. The data suggest that the expression of EIF3S3 is increased in prostate cancer, and that one of the mechanisms underlying the overexpression is the amplification of the gene.

Molecular cytogenetic analyses, especially comparative genomic hybridisation (CGH), have indicated that gain of the long arm of chromosome 8 (8q) is one of the most common chromosomal aberrations in many human malignancies, including breast and prostate carcinomas (Forozan *et al*, 1997). For example, about 70–90% of metastatic and/or hormone-refractory prostate carcinomas as well as about 50% of untreated breast cancers contain gain of 8q by CGH (Visakorpi *et al*, 1995; Cher *et al*, 1996; Tirkkonen *et al*. 1998; Nupponen *et al*, 1999). The gain of 8q has also been found to be associated with an aggressive phenotype and poor prognosis in these malignancies (Isola *et al*, 1995; Alers *et al*, 2000). Two minimal regions of amplification, 8q21 and 8q23–q24, have been identified in prostate cancer by CGH analyses (Cher *et al*, 1996; Nupponen *et al*, 1998). Several putative target genes, such as MYC, PSCA, EIF3S3 and TRPS1, have been proposed for the amplification of 8q23–24 (Jenkins *et al*, 1997; Reiter *et al*, 1998; Nupponen *et al*, 1999; Chang *et al*, 2000; Rummukainen *et al*. 2001). However, the significance of each of these genes in tumorigenesis is unclear.

MYC, located at 8q24.1, is a well characterised oncogene, which is involved in early embryogenesis, control of cell growth, cell differentiation and apoptosis (Giaccia and Denko, 2000). Its overexpression or amplification has been found in several cancer types, including breast and prostate cancer (Jenkins *et al*, 1997; Nupponen *et al*, 1998; Rummukainen *et al*, 2001).

We have recently identified two putative target genes for 8q amplification, EIF3S3 and TRPS1 (alias GC79) both located at 8q23 (Nupponen *et al*, 1999; Chang *et al*, 2000). Using suppression subtraction hybridisation (SSH), EIF3S3 was found to be overexpressed in breast cancer cell line SK-Br-3, which contains a high-level amplification at 8q23–q24 by CGH (Nupponen *et al*, 1999). Subsequently, it was found that the gene is amplified in about 30–50% of hormone-refractory, but only in 10–20% of untreated prostate carcinomas. The amplification of the gene has been found to be associated with high Gleason score (Saramäki *et al*, 2001). In addition, about 20% of untreated breast cancers contain amplification of EIF3S3 gene (Nupponen *et al*, 1999). The EIF3S3 gene encodes for a p40 subunit of eukaryotic translation initiation factor 3 (eIF3). The eIF3 complex plays a central role in the translation initiation pathway by binding to 40S ribosomal subunits in the absence of other translational components, and it helps to maintain the 40S and 60S subunits in a dissociated state (Asano *et al*, 1997). The function of the p40 subunit itself is, however, not known. It has been suggested that also some other translation initiation factors might be involved in the development of malignancies. For example, the overexpression of EIF4E as well as EIF4G1 has been shown to transform normal cells (De Benedetti and Rhoads, 1990; Fukuchi-Shimogori *et al*, 1997) and increased expression of EIF4E has been found in breast cancer cell lines (Anthony *et al*, 1996). In addition, a recent cDNA microarray study suggested that upregulation of several genes of translation apparatus is generally involved in metastasis of cancer (Ramaswamy *et al*, 2003).

TRPS1 was recently found to be more expressed in androgen-dependent than androgen-independent LNCaP prostate cancer cell lines by differential display analysis (Chang *et al*, 2000). TRPS1 encodes a zinc-finger GATA-type nuclear protein, which has been implicated in apoptosis. Castration leads to increased expression of TRPS1 in rat ventral prostate (Chang *et al*, 2000, 2002). Germ-line mutations in the TRPS1 gene, on the other hand, cause tricho-rhino-phalangeal syndrome (TRPS) types I and III (Momeni *et al*, 2000, Lüdecke *et al*, 2001). In addition, it was reported that xenopus TRPS1 acts as a repressor of the xenopus GATA4 transcription factor (Malik *et al*, 2001). This suggests that human TRPS1 could be involved in gene regulation of the family of human GATA transcription factors.

In order to evaluate the significance of EIF3S3, MYC, and TRPS1 in breast and prostate cancer, we have analysed the expression and copy number of the three genes in cell lines and tumours using quantitative real-time RT–PCR and fluorescence *in situ* hybridisation (FISH).

## MATERIALS AND METHODS

### Cell lines and tumour samples

Five prostate cancer cell lines (PC-3, LNCaP, DU145, 22rv1 and NCI-H660) and six breast cancer cell lines (SK-Br-3, ZR75-1, MCF-7, MDA436, EFM19 and T47D) were obtained from the American Type Culture Collection (ATCC, Manassas, VA, USA) and cultured under the recommended conditions. Freshly frozen specimens from nine benign prostate hyperplasias (BPH), 35 untreated primary (from 34 prostatectomies and one transurethral resection of the prostate) and 12 locally recurrent hormone-refractory (from transurethral resections of the prostate) prostate carcinomas were obtained from the Tampere University Hospital. The specimens were histologically examined for the presence of more than 60% of cancerous or hyperplastic tissue using haematoxylin and eosin-stained slides. The BPH samples were obtained from prostatectomy specimens from cancer patients. However, the specimens were histologically verified not to contain any cancer cells. The TNM distribution of the untreated cases was pT1N0M0, 2; pT2N0M0, 12; pT2N1M0, 2; pT3N0M0, 13; T4NXM0, 1; TXN0M0, 1; TXNXMX, 4. Of the untreated primary carcinomas, 11 were grade I, 16 grade II and seven grade III. Histological grade information was not available for one case. In the hormone-refractory cases, time from the beginning of the therapy to TURP varied from 15 to 60 months. In addition, a tissue microarray (TMA) containing 48 locally recurrent (TURP samples) hormone-refractory formalin-fixed paraffin-embedded prostate carcinomas from the Tampere University Hospital, was constructed according to previously published guidelines (Kononen *et al*, 1998). The use of clinical tumour material has been approved by the Ethical Committee of the Tampere University Hospital.

### RT–PCR

One to three 20 *μ*m frozen sections were cut using a cryotome. The total RNAs were isolated from the sections using Qiagen RNeasy MiniKit (Qiagen Inc., Valencia, CA, USA), and used for the first-strand cDNA synthesis with Superscript™ II reverse transcriptase and oligo d(T)_12–18_ primer according to the manufacturer's protocol (Life Technologies, Gaithersburg, MD, USA). For preparing the standard curve, 5 *μ*g of total RNA from the prostate cancer cell line LNCaP (ATCC, Manassas, VA, USA) was reverse transcribed as described above. After the first-strand cDNA synthesis, serial dilutions (1 : 5) were made to correspond to cDNA transcribed from 500, 100, 20, 4, 0.8 and 0.16 ng of total RNA. Primers for the TRPS1, EIF3S3 and MYC genes were designed with the Primer3 program (available at http://www-genome.wi.mit.edu/c gi-bin/primer3.www.cgi). To avoid amplification of any genomic DNA, the forward and reverse primers for each gene were chosen from different exons. The sizes of the PCR products were designed to be under 400 bp to optimise the RT–PCR measurements. The primer and probe sequences for the genes are given in Table 1

**Table 1:** Primer and probe sequences used in the real-time RT–PCR

**Gene**	**Primer sequences (5′–3′)**	Hybridization probe sequences (5′–3′)*
EIF3S3	GCC CAG GCT CTT CAA GAA TAC	GCTGAATCTCCCGAGCCGCCTTT-Fluorescein
	ATA GCC AAA ATC GGC AAT GA	Red640-CCTTTGCCTTTCCCTGCTGCGC

C-MYC	CCT ACC CTC TCA ACG ACA GC	GCCTCCCTCCACTCGGAAGGACT-Fluorescein
	CGC CTC TTG ACA TTC TCC TC	Red640-TCCTGCTGCCAAGAGGGTCAAGTT

TRPS1	GTA TCC TGC ATC GGG AGA AA	
	AGC TTC TGG TAG AGG CCA CA	

* purchased from Tib Mol Biol, Berlin, Germany.

.

**Figure 1:**
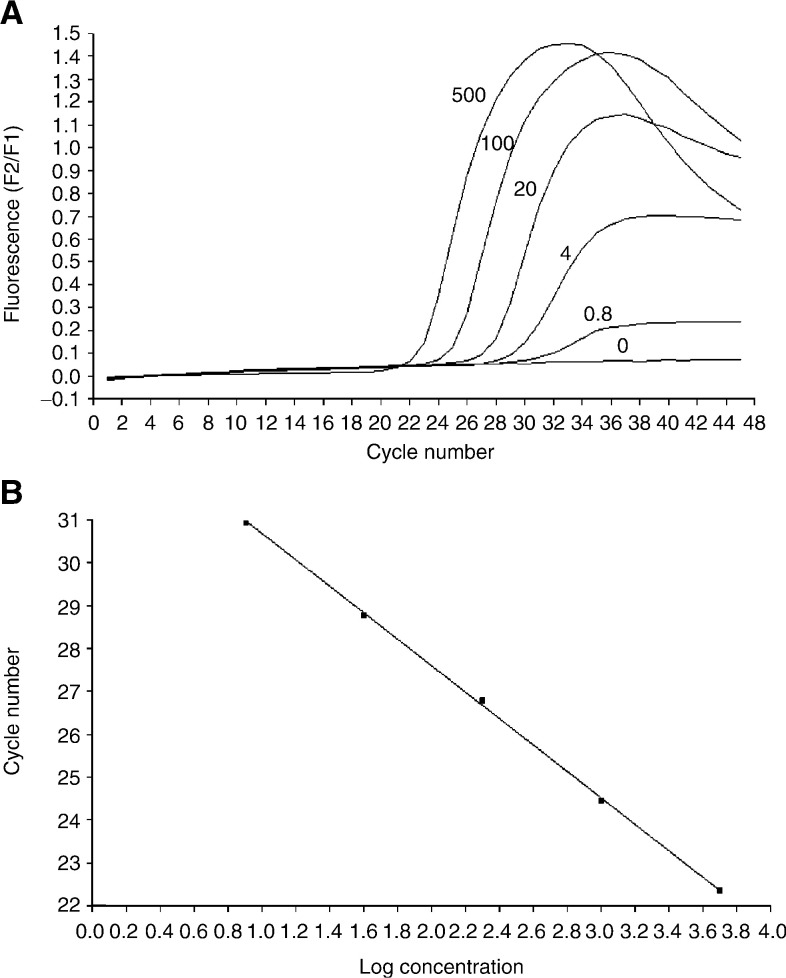
Standard curve of the MYC expression measurement by real-time RT–PCR. (**A**) Serially diluted standard corresponding to cDNA transcribed from 500, 100, 20, 4 and 0.8 ng of total RNA. Cycle number is blotted against fluorescent signal obtained in every cycle at the end of the annealing step. (**B**) Standard curve blotting cycle number at the crossing-point values of each standard against the logarithmic value of concentration of the standards.

The PCR reactions were performed with a LightCycler™ instrument using the LightCycler – FastStart DNA Master Hybridization Probes Kit (EIF3S3 and MYC) or FastStart DNA Master SYBR Green I Kit (TRPS1) (Roche Diagnostics, Mannheim, Germany). Thermocycling for each reaction was carried out in a final volume of 20 *μ*l containing 2 *μ*l of cDNA sample (or standard), 4 mM MgCl_2_, 0.5 *μ*M of each primer, 0.2 *μ*M of fluorescein and 0.4 *μ*M LC Red640 labelled probes (or SYBR Green I stain in TRPS1 assay), as well as 1 × ready-to-use reaction mix including Taq DNA polymerase, reaction buffer and dNTP mix. After 10 min of initial denaturation at +95°C, the cycling conditions of 50 cycles consisted of denaturation at +95°C for 1 s (EIF3S3 and MYC) or 10 s (TRPS1), annealing at +58°C for 10 s (EIF3S3 and MYC) or at +60°C for 7 s (TRPS1) and elongation at +72°C for 10 s (EIF3S3) or 13 s (MYC) or 7 s (TRPS1). After the PCR reaction and fluorescence measurements, fit point method together with background adjustment was used to determine the cycle in which the log-linear signal was distinguished from the background, and that cycle number was used as the crossing-point value. The software produced the standard curve by measuring the crossing point of each standard and plotting them against the logarithmic values of concentrations (Figure 1

). The expression levels of TRPS1, EIF3S3 and MYC were normalised by the expression level of the housekeeping gene TATA binding protein (TBP), measured as previously described (Linja *et al*, 2001). The relative expression was illustrated by dividing the EIF3S3, TRPS1 and MYC values with the TBP value, and multiplying by 10. TBP was chosen for the reference gene, because there are no known retropseudogenes for it and its expression is lower than that of many commonly used abundantly expressed reference genes (Bieche *et al*, 1999). After the PCR, every sample was also run in 1.2% agarose gel electrophoresis to ensure that the right size product was amplified in the reaction. Melting curve analysis was also used to evaluate the quality of the PCR reaction for TRPS1.

### Fluorescence *in situ* hybridisation

Locus-specific PAC probes for human TRPS1 (Chang *et al*, 2000), EIF3S3 (Nupponen *et al*, 1999) and MYC (Nupponen *et al*, 1998) were labelled with digoxigenin-dUTP (Roche Diagnostics), and a pericentromeric probe for chromosome 8 (pJM128) with FITC-dUTP (NEN, Boston, MA, USA), by nick translation. The metaphase preparations from the cancer cell lines were prepared using standard techniques. The dual-colour hybridisation was performed essentially as described previously (Hyytinen *et al*, 1994). Briefly, the slides were denatured in a 70% formamide/2 × SSC solution (pH 7.0) at 70°C for 3 min and dehydrated in an ascending ethanol series. Hybridisation was performed over two nights at 37°C. After stringent washes, the slides were stained with antidigoxigenin-rhodamine (Roche Diagnostics) and counterstained with an antifade solution (Vectashied, Vector Laboratories, Burlingame, CA, USA) containing 4,6-diamidino-2-phenylindole (DAPI). The formalin-fixed paraffin-embedded TMAs were pretreated and hybridised as described previously (Saramäki *et al*, 2001). The FISH signals were scored from nonoverlapping epithelial cells using Olympus BX50 epifluorescence microscope (Tokyo, Japan). Tumours with a tight cluster of signals or at least two-fold higher copy number of the locus-specific probe signals *vs* centromeric signals or ⩾5 copies of locus specific probe signals were considered to contain a high-level amplification of either gene. Tumours with three to four copies of the gene signals were considered to have a gain of the gene.

### Statistical analyses

The associations of the gene copy numbers, tumour types, histological grades and clinical stages with expression levels were calculated with nonparametric Kruskal–Wallis and Mann–Whitney *U*-tests. Outliers were detected by using extreme studentised deviate method (ESD).

## RESULTS

### TRPS1, EIF3S3 and MYC gene amplification

Tissue microarrays and FISH were first used to study gene copy number of EIF3S3 and TRPS1 in hormone-refractory prostate carcinomas. High-level amplification (⩾5 copies) of EIF3S3 and TRPS1 was found in 11out of 40 (28%) and 10 out of 36 (28%) of the cases, respectively. The gain (three to four copies) of EIF3S3 and TRPS1 was found in 19 out of 40 (48%) and 18 out of 36 (50%) of the cases, respectively. In the cases of high-level amplification, the genes were always coamplified. The coamplification was also verified by hybridizing differentially-labelled gene specific probes (biotin labelled EIF3S3 probe and AlexaFluor 594 labelled TRPS1 probe) simultaneously to the TMA.

**Figure 2:**
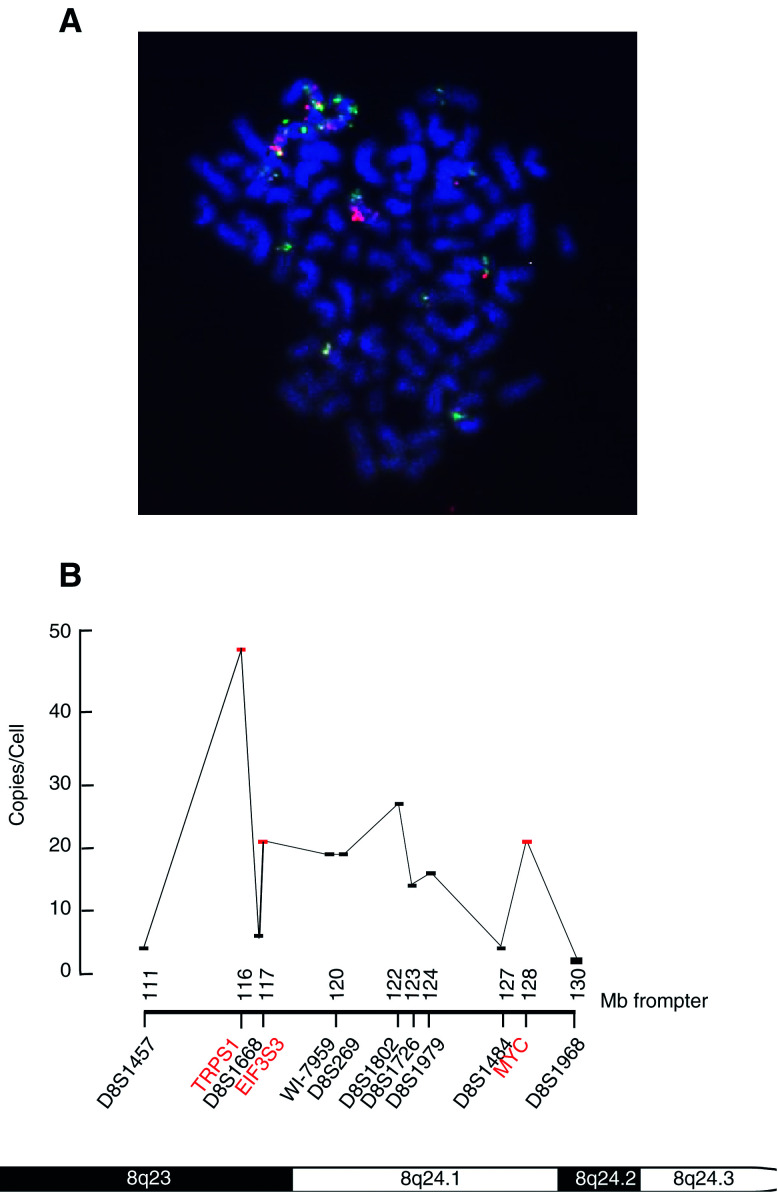
(**A**) Dual colour FISH with PAC probes for EIF3S3 (green) and TRPS1 (red) in metaphase preparation of breast cancer cell line SK-Br-3. Multiple copies of both genes are seen in marker chromosomes. (**B**) Copy numbers of TRPS1, EIF3S3, MYC and anonymous sequence tag sites (STSs) in SK-Br-3 breast cancer cell line by FISH. The copy numbers of the STSs are from a previous publication (Nupponen *et al*, 2000). At the bottom, the order and distances in megabases (Mb) of the genes (marked in red) and STSs (marked in black) from the p-telomere (pter) of the chromosome 8 are shown.

Next, breast and prostate cancer cell lines were analysed for copy numbers of the EIF3S3, MYC and TRPS1 genes. Of the cell lines, the highest copy number of all the genes was found in the breast cancer cell line SK-Br-3, which showed 47 copies of TRPS1 and 21 copies of EIF3S3 and MYC and only one copy of chromosome 8 centromere (Figures 2

and 3

**Figure 3:**
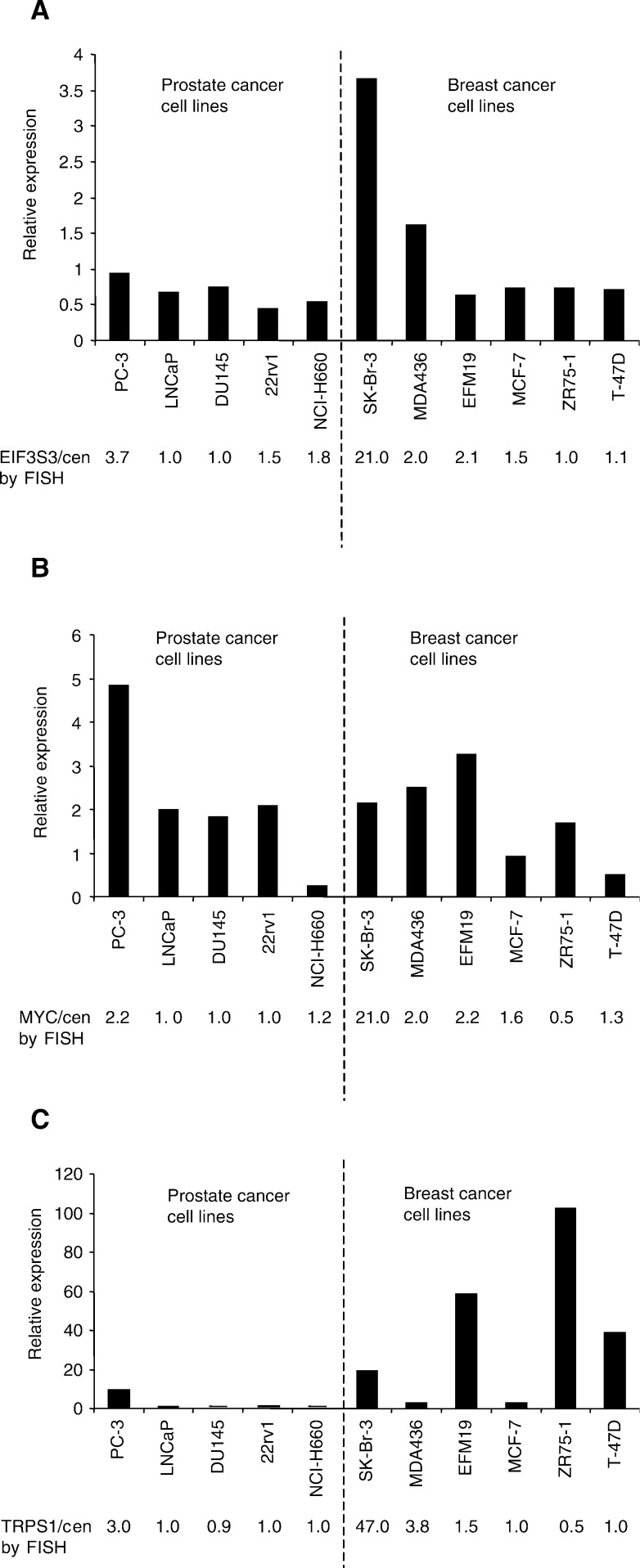
Relative expression and gene copy number of (**A**) EIF3S3, (**B**) MYC and (**C**) TRPS1 in prostate and breast cancer cell lines by real-time quantitative RT–PCR and FISH. The relative expression of the genes was calculated by dividing the expression value of the gene of interest with the expression value of housekeeping gene TBP. The relative gene copy number was calculated by dividing the signal copy number of the gene of interest with the signal copy number of chromosome 8 centromere. Of the cell lines, SK-Br-3 showed clearly the highest copy number of all genes. However, only EIF3S3 was overexpressed in the cell line.

). In addition, a high-level amplification (locus/centromere ratio ⩾2) of all three genes was found in MDA436 and PC-3 cancer cell lines. EIF3S3 and MYC were highly amplified also in EFM19 (Figure 3).

### Expression of TRPS1, EIF3S3 and MYC

The standard curves in the real-time quantitative RT–PCR assay showed wide dynamic range and the linear relationship between cycle number and fluorescent threshold was strong (*r*^2^∼1). In addition to TBP, the expression of *β*-actin was measured and used alternatively for normalisation of most of the samples (data not shown). The results were similar with both control genes. Due to the potential problems with the *β*-actin retropseudogenes, TBP was chosen for normalisation of the whole material.

Figure 3 illustrates the relative expression of TRPS1, EIF3S3 and MYC in breast and prostate cancer cell lines. In the breast cancer cell line SK-Br-3, which contains the highest copy number of all the three genes investigated, EIF3S3 was the only gene that showed remarkably high-level (three-to 10-fold compared to the other cell lines) overexpression. The relative expression of TRPS1 was highest in ZR75-1, which contains a loss of the gene compared to centromere copy number. Interestingly, in most breast cancer cell lines, the expression of TRPS1 was high compared to the expression levels of EIF3S3 and MYC. The high expression level of TRPS1 in the breast cancer cell lines has also been observed by Northern and Western blot analyses (Chang *et al*, unpublished).

Figure 4

**Figure 4:**
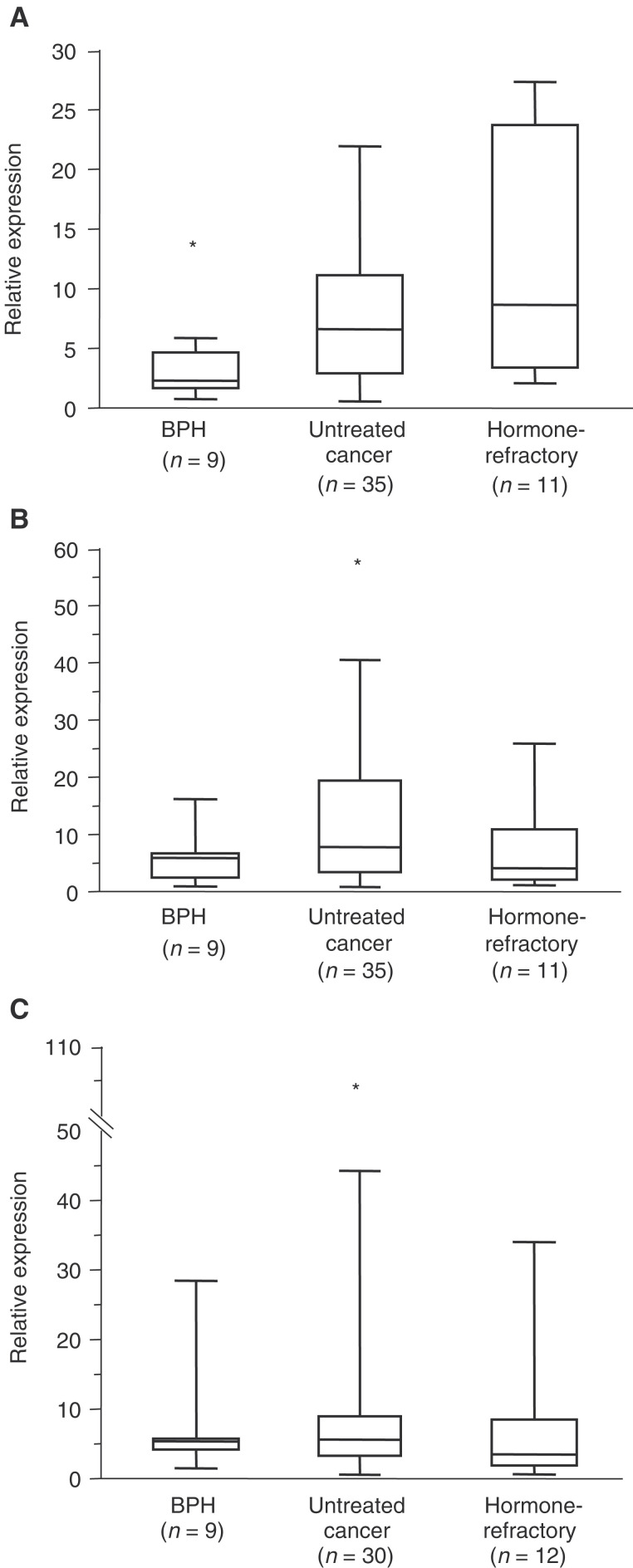
Box and whisker plots displaying the relative expression of (**A**) EIF3S3, (**B**) MYC and (**C**) TRPS1 in prostate tumour samples analysed by real-time quantitative RT–PCR. The boxes indicate the area of 50% of samples, the horizontal line in the boxes indicates median value. The whiskers display the range. Stars depict the outlier values as estimated by extreme studentised deviate method. The expression of EIF3S3 was significantly (*P*=0.029) higher in carcinomas than in BPH. There were no significant differences in the expression levels of TRPS1 and MYC between the tumour groups.

illustrates the relative expression of TRPS1, EIF3S3 and MYC in BPH (*n*=9), untreated primary (*n*=30 for TRPS1, *n*=35 for EIF3S3 and MYC) and locally recurrent hormone-refractory (*n*=12 for TRPS1, *n*=11 for EIF3S3 and MYC) prostate carcinomas. The expression of EIF3S3 was, on average, three-fold higher (*P*=0.029) in carcinomas than in BPH. There was no difference in the level of expression of EIF3S3 in hormone-refractory and untreated prostate carcinomas. MYC and TRPS1 were expressed in equal levels in BPH, untreated and hormone-refractory tumours. There were no significant associations between histological grade or clinical stage (T3–T4 and/or N+ and/or M+ *vs* T1-2N0M0) and the expression of any of the three genes in the untreated tumours.

## DISCUSSION

Several oncogenes are activated by overexpression of the gene and one mechanism of the overexpression is amplification of the gene (Brodeur and Hogarty, 1998). Gain or amplification of chromosome 8q is one of the most common chromosomal alterations in breast and prostate cancer (Forozan *et al*, 1997). However, the target gene of the amplification is still unknown. The genes studied here, EIF3S3, TRPS1 and MYC, have been suggested to be putative target genes in 8q23–q24 (Jenkins *et al*, 1997; Nupponen *et al*, 1999; Chang *et al*, 2000). In order to evaluate the significance of these genes in breast and prostate cancer, we analysed both the gene copy numbers as well as the expressions of the three genes.

We have previously shown that EIF3S3 and MYC are coamplified in about one-third of the locally recurrent hormone-refractory prostate carcinomas (Saramäki *et al*, 2001). Now, it was found that also EIF3S3 and TRPS1 are coamplified in about 30% of the hormone-refractory prostate tumours. The finding that all three genes are commonly coamplified in the hormone-refractory tumours indicates that the size of the amplicon is large. The TRPS1 and EIF3S3 genes are located about 12 and 11 Mb centromeric from MYC, respectively. The large size and the relatively low copy number of the amplicon have previously been implicated also by CGH and FISH studies (Visakorpi *et al*, 1995; Cher *et al*, 1996; Nupponen *et al*, 1998, 2000).

The majority of cancer cell lines have been established from metastatic lesions of cancer. They typically contain more chromosomal alterations than primary tumours, and the aberrations are more confined. For example, amplicons are often smaller and the copy numbers higher making the cell lines more informative than the primary tumours for mapping the amplicons (Kallioniemi *et al*, 1994). Of the breast and prostate cancer cell lines, however, only SK-Br-3 shows a high-level amplification of two (8q21 and 8q23–24) independent subarm regions by CGH (Kallioniemi *et al*, 1994; Nupponen *et al*, 1999). Of the three genes analysed here, TRPS1 showed the highest copy number, about twice as high as for either EIF3S3 or MYC in SK-Br-3. Together with our previous mapping data (Nupponen *et al*, 2000), the results indicate that SK-Br-3 contains high copy numbers of all the three genes with low copy numbers of the flanking regions (Figure 1B). Thus, the amplicon from TRPS1 to MYC, covering a chromosomal region of about 12 Mb, contains, at least, three independent subamplicons in SK-Br-3.

We used real-time quantitative RT–PCR approach to measure the expression of TRPS1, EIF3S3 and MYC. In real-time PCR, the quantification of the template is based on detection of the cycle in which the reaction enters the exponential phase, instead of measuring the amount of end product. Thus, none of the reagents is rate limiting in the reaction at the time of measurement of the fluorescence. Several studies have already shown that real-time RT–PCR is a highly quantitative and reliable method (Bieche *et al*, 1999; Linja *et al*, 2001; Savinainen *et al*, 2002). It is also especially useful in analysis of small tumour samples.

We first analysed the expression of EIF3S3, MYC and TRPS1 in the cell lines. In SK-Br-3, which showed the highest copy number of the genes, the expression of only EIF3S3 was remarkably high. The high-level expression of EIF3S3, shown here by quantitative RT–PCR, confirms our earlier Northern blot data (Nupponen *et al*, 1999). Surprisingly, the expression of TRPS1, whose copy number was highest of the three genes in SK-Br-3, was lower in SK-Br-3 compared to ZR75-1. The expression of TRPS1 was the highest in ZR75-1, which actually contains a relative loss of the gene. The expression of MYC varied among the cell lines. SK-Br-3 containing the highest copy number of the gene did not show clearly increased expression compared to the other cell lines. The data suggest that of the three genes, EIF3S3 is the most likely target gene of amplification in SK-Br-3.

In the clinical prostate cancer specimens, the level of EIF3S3 expression was significantly (*P*=0.029) higher in prostate cancer than in BPH. The data are consistent with our previous analyses by semiquantitative mRNA *in situ* hybridisation, which suggested that EIF3S3 is expressed more in hormone-refractory prostate carcinomas than in BPH (Nupponen *et al*, 1999). Here, it was found that the expression of EIF3S3 is increased also in untreated prostate cancers. Somewhat surprising, the expression of EIF3S3 was not higher in the hormone-refractory compared to the untreated tumours, despite the fact that hormone-refractory tumours, in general, contain higher frequency of 8q gain (Visakorpi *et al*, 1995). The data suggest that EIF3S3 is commonly overexpressed in prostate cancer, and also other mechanisms than gene amplification may lead to the overexpression of the gene. There were no significant differences between the expression of either MYC or TRPS1 in BPH, untreated and hormone-refractory carcinomas, suggesting that alterations in the expression of these two genes are not generally involved in the progression of prostate cancer.

In conclusion, the results indicate that overexpression of MYC and TRPS1 are rare in prostate cancer *in vivo*. In contrast, the expression of EIF3S3 is increased in prostate cancer. One mechanism for the overexpression of EIF3S3 seems to be amplification of the gene as demonstrated by the cell line SK-Br-3. Analyses of the expression levels of EIF3S3 in large clinical materials are now warranted.
